# Prevalence of Adolescents’ Persistent High Utilization of Outpatient Healthcare Services and ICD-10 Diagnoses: A Retrospective 4-Year Population-Based Register Study

**DOI:** 10.1177/21501319261421476

**Published:** 2026-02-21

**Authors:** Pipsa Lahtinen, Mika Niemelä, Helinä Hakko, Sanni Penttilä, Petri Kivinen, Sami Räsänen

**Affiliations:** 1 University of Oulu, Finland; 2 Oulu University Hospital, Finland; 3 Central Finland Wellbeing Services County, Jyväskylä, Finland

**Keywords:** children, adolescent, persistent high utilizer, ICD-10, healthcare service use

## Abstract

**Background:**

Persistent high healthcare utilization has rarely been analyzed in adolescent populations although there are specific chronic health issues also among adolescents, like mental health challenges and some somatic illnesses, which need long-term treatments. Therefore, it remains unclear whether such high utilization is persistent among adolescents. Recognizing patterns of high service utilization and its’ persistence are essential for preventing avoidable healthcare use.

**Materials and Methods:**

This population-based study focused on all adolescents born in 2004 (n = 1483) from North Karelia, Finland. Healthcare service use data (2018-2021) was extracted from the electronic patient register. A high utilizer of healthcare (HU: the abbreviation also refers to high utilization) was defined as a person having been in contact with healthcare services during at least 12 days a year (attendance days). A persistent HU (pHU) was a person identified as being HU during 3 or 4 of all 4 follow-up years. The prevalence of diagnoses set to young people was compared in accordance with ICD-10 diagnostic categories between pHU and non-pHU groups.

**Results:**

pHUs accounted for 18.5% (n = 275) of all adolescents born in 2004. A total of 53.5% (n = 793) of adolescents met the criteria of single-year HU during the follow-up period, and of these, 34.7% were also identified as pHU. The pHU group was particularly emphasized in mental and behavioral disorders (RR = 4.5, CI: 3.6-5.5, *P* < .001), as well as the diseases of the nervous system (RR = 3.4, CI: 2.2-5.1, *P* < .001) and musculoskeletal system/connective tissue (RR = 3.2, CI: 2.4-4.3, *P* < .001).

**Conclusions:**

The continuation from single-year to multi-year HU was relatively high. Thus, single-year high utilization is a strong indicator of future persistence in healthcare service utilization. Further research is needed to identify differences in clinical and psychosocial characteristics between single-year high utilizers and those whose high-utilization continues for years.

## Introduction

High utilization (HU) of healthcare services is an essential phenomenon in society, healthcare, and research.^32^ For example, HU is reported to cause substantial costs for organizing healthcare service systems.^12,24,35^ Previous studies on HU have explored the topic by analyzing either accumulated costs or the number of healthcare visits that have taken place within a fixed time-period. The HU populations via expenditures are frequently referred to as high-cost users or patients.^7,22,25^ Likewise, HU patients defined through the high number of healthcare visits have been identified as frequent attenders (FA),^2,29^ frequent users,^16,19^ high users,^3,31^ or high utilizers (HUs).^20^ When defining HU populations, the most commonly used cut-off points for the number of visits or cost amounts are set to the upper percentiles of distribution, such as 1%, 5%, and 10%.^5,23^ Furthermore, in some studies high utilization has been defined by setting the absolute frequency of visits, an HU being a person who has had 10 visits per year.^21,26^

Previous research on HU has concentrated on elderly people,^4^ while HU studies on children and adolescents are rare. The research on the population aged 0 to 24 years reported that 5% of this age group accounted for 54% of total annual costs in primary and secondary healthcare services.^22^ In the study on 7- to 12-year-old children in primary care, 21% accounted for 58% of single-year attendances.^11^ HU with respect to young people has commonly been related to chronic conditions^1^ such as mental health, as well as respiratory, neurological, and metabolic conditions.^12,22^

While only a limited number of research articles on HU in adolescent populations are published, the number of studies that have specifically analyzed consistency in high levels of service utilization that last longer than 1 year is even less.^1^ The study on adult patients^22^ explored HU (top 10%) and its persistence by utilizing 8-year healthcare registry data. They showed that 4.1% of HU persons of healthcare services in any year were also HUs in the subsequent year. In a Finnish 6-year follow-up study of adult service utilization, 6.6% of FAs (ie, having ≥10 visits per year) were also FAs for 3 consecutive years and 1.1% for 5 years.^26^ A study of young patients aged below 19 years^27^ analyzed service use in 3 time periods from 1999 to 2011. They reported that older adolescents aged 17 to 19 years persisted most likely in the highest percentile (top 10%) for 3 or more years.

Additional research on HU persistence respective to healthcare services among young people is needed for several reasons. In particular, research focusing on the use of outpatient healthcare services is necessary, because these services are responsible for both first-line treatment and long-term treatment of chronic health conditions. In addition, more studies with several-year follow-up periods are required, as the annual turnover rate, that is, shifts from HU to non-HU groups, or vice versa, is reported to be quite high.^20,32^ Recognizing the conditions causing long-term need and use for healthcare services is crucial, because such conditions can be risk factors for a child’s normal physical and mental growth.^8^ Research-based information on pHU in young people would be useful for the early identification of potential individuals with continuous health problems and for developing healthcare services to respond to their needs.

The present study analyses high utilization (HU) and its persistence in outpatient healthcare services among the entire adolescent population born in 2004 from the wellbeing services county of North Karelia, Finland. A single-year HU was defined as based on the frequency of annual attendance days (ie, face-to-face visits and other types of registered contacts) to health care services (see further details in *Materials and Methods* section). Persistence of HU (pHU) was defined as belonging to an HU group during 3 to 4 of all follow-up years (2018-2021). In the first phase, the annual attendance days of each adolescent were calculated to identify the cut-off value for the number of attendance days needed to define single- and multiple-year HUs as well as pHU and non-pHU adolescent groups. The subsequent subgroup analysis focused on the attendance days that included ICD-10 diagnosis as defined by the treating physician. The prevalence of illnesses according to the main ICD-10 diagnostic categories was compared between pHU and non-pHU adolescent groups.

## Materials and Methods

### Study Design

The study is part of the Finnish North Karelia collective impact (NKCI)—study project. The target population is comprised of all children and adolescents aged below 18 years living in North Karelia (referred to as *Siun Sote*) county and who have the right to access healthcare services in the Wellbeing service county. The register-based data includes comprehensive information on the utilization of healthcare and social services recorded in the electronic client and patient system (Mediatri) of *Siun Sote.* In Finland, wellbeing services counties are funded by the state, and they are responsible for organizing and providing health care services for the inhabitants of municipalities belonging to these counties. All Finnish citizens enjoy equal access to both primary and secondary level healthcare services.

The North Karelia region has used a unified patient information system since 2011, which has enabled register-based research covering the entire region. In terms of both its scope and the length of the accumulated historical data, it is an exception even within Finland. What makes it particularly distinctive is that, since 2017, social welfare records have also been available under the same data controller and as region-wide service data. In the eastern part of Finland, North Karelia region, an administrative integration of all levels of health and social services was already carried out in 2017 aiming to deliver care that is more patient-centered, effective and efficient, and improving health outcomes while maximizing resource use.^31^ In practice, service integration in North Karelia meant administrative integration, including functional integration of electronic health record system in all health services across the region.

When the Joint Municipal Authority for North Karelia Social and Health Services (Siun sote) was founded, it brought together the health service organizations of 14 municipalities.^33^ The reform created a single structure that merged social and health care functions, including secondary-level services, under 1 management and unified financial and resource administration. Siun sote’s service network consisted of a central hospital and 22 health stations, employing roughly 400 physicians, 4900 nurses and other health care professionals, as well as about 2100 additional staff such as social workers, rescue personnel, technical staff, assistants, and administrative employees.

Permission to use register data was received from the Joint Municipal Authority for North Karelia Social and Health Services (nowadays *wellbeing services county of North Karelia*; DNo. 101/13.00.01.01/2017, granted on February 7, 2024).

### Study Sample of Adolescents Born in 2004

The target population interest comprised of all adolescents born in 2004 and who had lived in North Karelia during the whole 2018 to 2021 study period. It included 1483 adolescents aged 17 years at the end of 2021. Of them 734 (49.5%) were males and 749 (50.5%) females. According to statistical grouping of municipalities by Statistics Finland^30^, 814 (54.9%) adolescents were from urban region, 346 (23.3%) from semi-urban area, and 323 (21.8%) from rural area.

### Definition for HU and pHU

The data on the use of healthcare services during the period 2018 to 2021 was collected from the Mediatri. The registry data includes information on all visits and contacts to primary and special healthcare settings. Physicians set the diagnosis of patient by using ICD-10 diagnostic categorization and record it on patient register according to ICD-10 codes, while other health care professionals, like nurses, records reasons for visits according to the International Classification of Primary Care, Second Edition (ICPC-2).^37^

The definition for high utilizer (HU) of healthcare was based on the annual number of attendance days in primary and specialized healthcare services. ‘Annual attendance days’ refers to the number of days a person has had face-to-face visits or other types of registered contacts such as phone calls or virtual contacts within the context of healthcare services during a calendar year.

The number of attendance days at the upper quartile (75th percentile) was selected as the annual cut-off value to indicate high healthcare use by adolescents. By applying this statistical cut-off value, an adolescent was defined to be single-year HU if s/he has had 12 or more attendance days per calendar year (see Supplemental Table 1). Furthermore, a study participant was defined to be a persistent HU (pHU) if s/he belonged to the HU group for at least 3 out of all 4 follow-up years.

### Service Use by Main ICD-10 Diagnostic Categories

A subgroup analysis focused on the attendance days, which included ICD-10 diagnoses as primary diagnosis for a visit set by physicians using specified diagnostic criteria. In Finland, the International Classification of Diseases, 10th revision (ICD-10) from 1996 onwards is used as an official diagnostic classification system.^36^

If a patient’s attendance day included more than 1 contact with several healthcare professionals during the same day, the selection of a record with ICD-10 diagnosis for analysis was carried out as follows:

- the diagnosis recorded in connection with the physician’s appointment took precedence over those marked on visits to all other healthcare professionals.
- if a patient had several physician visits during a day, the selection of diagnosis for analysis was based on the following hierarchy: specialist/senior physician, chief physician, and health center physician. Exception: a disease-specific ICD-10 diagnosis set by a physician was prioritized instead of Z-codes (factors influencing health status and contact with health services).
- if a patient had several visits during a day with other healthcare professionals but no visits with a physician, the ICD-10 diagnosis marked on a visit with psychologists was preferred to a diagnosis recorded during a visit with a nurse.

After identifying the primary ICD-10 diagnosis recorded on annual attendance days of adolescents (n = 1483), the prevalence of illnesses by the main ICD-10 categories over the whole study period 2018 to 2021 was calculated. An adolescent was defined to belong to a certain illness category if at least 1 attendance day during the whole study period of 2018 to 2021 included an ICD-10 diagnostic code belonging to that illness category.

### Statistical Methods

The registry data used in analyses is based on guidelines provided for the wellbeing services counties in Finland by the Finnish Institute for Health and Welfare (THL).^9^ The permission and the data extracted for the use of the current study follows with THL’s information security policy (THL/957/3.01.00/2011), the General Data Protection Regulation [GDPR]), and CSC sensitive data (SD) guidelines for processing social and health data under the Finnish Act on Secondary Use of Social and Health Data.

The statistical significance of group differences in categorical variables was analyzed with a Chi-square or Fisher Exact test, and in continuous variables with a Student’s *t*-test or Mann-Whitney *U*-test. Rate ratio (RR) with 95% CI of the illnesses over the whole study period 2018 to 2021 was used to compare the prevalence rate of diseases defined by ICD 10-based main categories of diseases and conditions, between the study groups (pHU vs non-pHU). The statistical software used in the analyses was IBM Statistics, version 29.

## Results

### High Healthcare Use and Its Persistence

**Figure 1.**
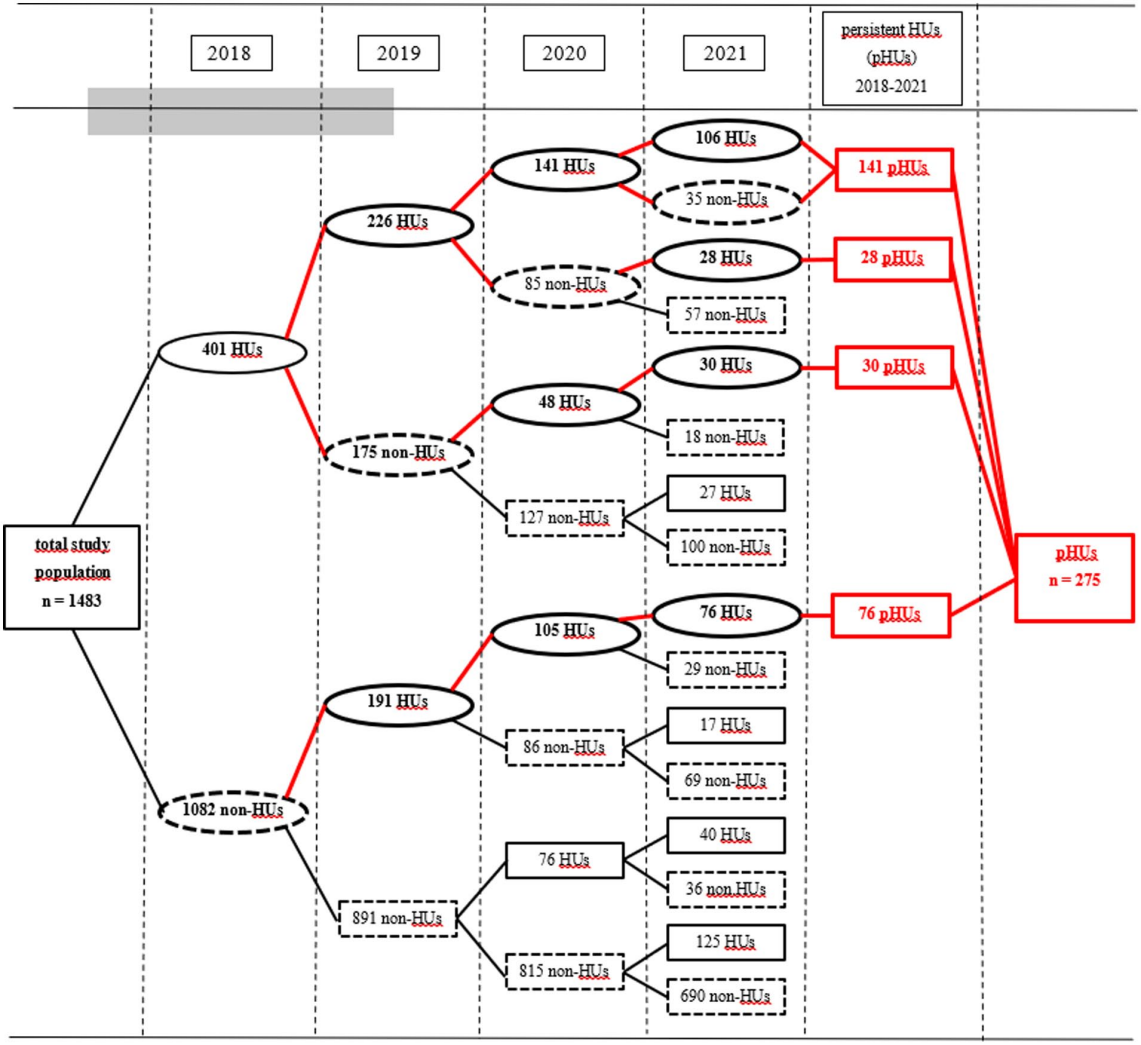
Pathway/continuation from single-year high healthcare utilizer (HU) to persistent HU (pHU) during the 4-year follow-up period among adolescents born in 2004.

**Figure 2.**
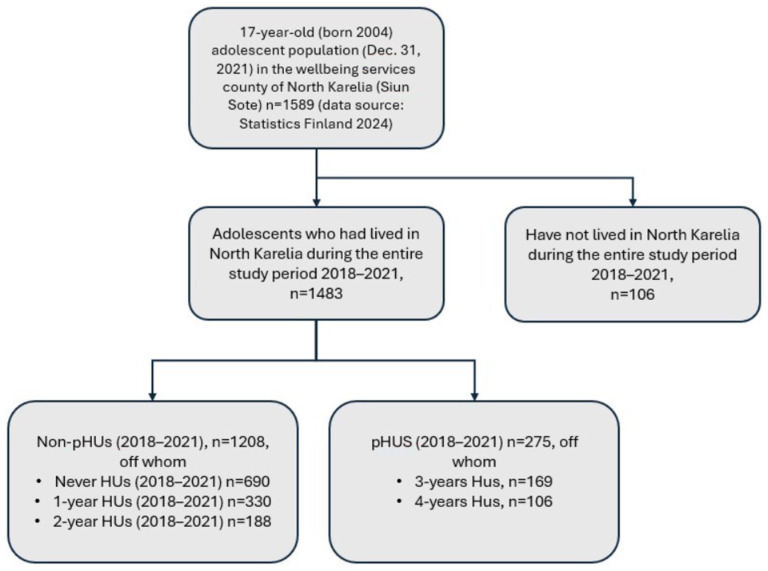
Study groups of persistent and non-persistent high healthcare utilizers among adolescents born in 2004.

Figure 1 shows the pathway from a single-year HU to multiple-year HUs over the 4-year study period among adolescents born in 2004 (n = 1483). Of the total sample of the adolescents, 275 (18.5%) met the criteria for being persistent HU during the 4-year follow-up period. As Figure 2 shows, 46.5% (n = 690) of the adolescents did not belong to the HU group in any of the follow-up years. Single-year HUs accounted for 793 (53.5%) of the total sample, and continuation to persistent HU made up 34.7% of these single-year HUs.

### Attendance Days With Recorded ICD-10 Diagnosis

Further subgroup analysis was focused on attendance days with recorded IC10-diagnosis, which were analysed by categories to ICD-10 main diagnostic categories and compared between the pHU- and non-pHU groups of adolescents (Table 1). In the total sample of adolescents, the most prevailing diagnostic categories were ‘Factors influencing health status and contact with services’ (95.8%) and ‘Diseases of digestive system, incl. dental visits’ (72.4%), followed by ‘Injury, poisoning, and certain other consequences of external causes’ (35.1%); ‘Symptoms, signs, and abnormal clinical and laboratory findings, not elsewhere classified’ (30.5%); ‘Diseases of the respiratory system’ (26.9%); and ‘Mental and behavioral disorders’ (26.6%). When comparing the prevalences of illnesses between the pHU- and non-pHU groups of adolescents, the highest RRs were observed in categories for ‘Mental and behavioral disorders’ (RR = 4.46, CI: 3.64-5.46, *P* < .001); ‘Diseases of the nervous system’ (RR = 3.39, CI: 2.23-5.10, *P* < .001); ‘musculoskeletal system and connective tissue’ (RR = 3.23, CI: 2.44-4.27, *P* < .001); ‘genitourinary system’ (RR = 3.22, CI: 2.39-4.34, *P* < .001); ‘neoplasms and diseases of the blood and blood-forming organs and certain disorders involving the immune system’ (RR = 3.17, CI: 1.93-5.14, *P* < .001); ‘endocrine, nutritional, and metabolic diseases’ (RR = 2.58, CI: 1.63-4.00, *P* < .001); and ‘symptoms, signs, etc.’ (RR = 2.45, CI: 2.01-2.98, *P* < .001).

**Table 1.** Prevalence of Illnesses Over the Whole Study Period 2018 to 2021 Among the pHU- and Non-pHU Groups of Adolescents Born in 2004, According to the ICD-10 Main Categories.

ICD-10 main categories of illnesses	Number of adolescents born in 2004			Rate ratio	*P*-value
	Total (n = 1483) (%)	Persistent HU, pHU (n = 275) (%)	Non-pHU (n = 1208) (%)		
Mental and behavioral disorders (F-codes)	395 (26.6)	199 (72.4)	196 (16.2)	4.46 (3.64-5.46)	<.001
Diseases of the nervous system (G-codes)	101 (6.8)	44 (16.0)	57 (4.7)	3.39 (2.23-5.10)	<.001
Diseases of the musculoskeletal system and connective tissue (M-codes)	215 (14.6)	92 (33.5)	125 (10.3)	3.23 (2.44-4.27)	<.001
Diseases of the genitourinary system (N-codes)	189 (12.7)	80 (29.1)	109 (9.0)	3.22 (2.39-4.34)	<.001
Neoplasms, Diseases of the blood and blood-forming organs, and certain disorders involving the immune mechanism (C-D-codes)	74 (5.0)	31 (11.3)	43 (3.6)	3.17 (1.93-5.14)	<.001
Endocrine, nutritional, and metabolic diseases (E-codes)	92 (6.2)	34 (12.4)	58 (4.8)	2.58 (1.63-4.00)	<.001
Symptoms, signs, and abnormal clinical and laboratory findings not elsewhere classified (R-codes)	452 (30.5)	162 (58.9)	290 (24.0)	2.45 (2.01-2.98)	<.001
Diseases of the circulatory system (I-codes)	36 (2.4)	11 (4.0)	25 (2.1)	1.93 (0.86-4.07)	.082
Certain infectious and parasitic diseases (A-B -codes)	125 (8.4)	37 (13.4)	88 (7.3)	1.85 (1.22-2.74)	.003
Diseases of the respiratory system (J-codes)	399 (26.9)	116 (42.4)	283 (23.4)	1.80 (1.44-2.24)	<.001
Diseases of the eye and adnexa, and Diseases of the ear and mastoid process (H-codes)	287 (19.4)	80 (29.1)	207 (17.1)	1.70 (1.29-2.20)	<.001
Injury, poisoning, and certain other consequences of external causes (S-T- codes)	520 (35.1)	135 (49.1)	385 (31.8)	1.54 (1.26-1.88)	<.001
Diseases of the skin and subcutaneous tissue (L-codes)	300 (20.2)	78 (28.4)	222 (18.4)	1.52 (1.18-2.01)	.001
Congenital malformations, deformations, and chromosomal abnormalities (Q-codes)	32 (2.2)	8 (2.9)	24 (2.0)	1.46 (0.57-3.37)	.354
Diseases of the digestive system (K-codes)	1073 (72.4)	236 (85.8)	837 (69.3)	1.24 (1.07-1.43)	.004
Factors influencing health status and contact with health services (Z-codes)	1419 (95.7)	272 (98.9)	1147 (95.0)	1.04 (0.91-1.19)	.541
Codes for special purposes (U-codes)	30 (2.0)	6 (2.2)	24 (2.0)	1.10 (0.36-2.75)	.807

Rate ratios (RR) are organized by the magnitude of the comparison between the rate of an outcome in the pHU group and the non-pHU group, with values greater than 1 indicating higher rate in the pHU group. A subgroup analysis focused on the attendance days, which included a ICD-10 diagnostic code recorded as primary diagnosis for a visit.

## Discussion

High healthcare service use and its continuation to persistent high utilization has been only sparsely studied in adolescent populations. In this study, we analyzed the continuity of high healthcare use (HU) in population-based data concerning adolescents born in 2004 from age 14 to 17 years. In this study, persistent HUs of healthcare (pHUs) were defined to be those adolescents who had 12 or more attendance days in healthcare services during a period of 3 or 4 years out of the total 4-year follow-up period.

The main finding of our study was that almost every fifth (18.5%) of the total adolescent population born in 2004 fulfilled the criteria for being pHU. Every second adolescent (53.5%) met the criteria of being a single-year HU for at least 1 year during the follow-up period, and 34.7% of them went on to assume persistent HU status. Comparing our results to previous studies is challenging, due to variations in study populations, statistical units, and the definition of HU. In earlier studies, the percentile cutoff for visits or costs defining persistent HUs has generally been set higher (the top 1%-10% of all users) compared to our study, in which the cut-off point was set at the upper quartile of annual attendance days. However, when defining HU group of participants, the annual number of visits (approximately 10 visits or more per year) in earlier studies^21,23,26^ has been very similar, with a total of 12 or more attendance days per year observed in our study. Analyzing high utilization by using absolute annual numbers is useful, because they are directly comparable and are also easy to relate to clinical practices as well as to the need for appropriate resources of services.

The finding of our research showing that one-fifth of adolescents use, on average, these services once a month or more is significant from the point of view of the service system, and can be considered as a burden on health care. In our study, the cut-off point of 12 annual attendance days for pHU clearly exceeds the internationally presented average numbers of visits generally in healthcare. According to the statistics of WHO European Region,^38^ the average number of outpatient visits in 2014 was 7.6 and, in Finland, 7.0 per person per year.^10^ The difference is clear, even though average numbers also include older populations who obviously experience more morbidity and also engage in more service use. Therefore, it would be important to study in child and adolescent populations the reasons for the transition from single-year to multiple-year HUs. This information would be valuable from the perspective of considering whether there can be identified potential targets for preventive interventions and possible deficiencies in the service system in the care of these patients or deficiencies in service systems.

When comparing the findings of our study to studies analyzing persistent use of health care in adult populations, the proportion of the child and adolescent population with pHUs can be considered high. For example, in a Finnish adult population study,^26^ 6.6% were HUs who used healthcare services during 3 and 1.1% during 5 follow-up years. In Ng et al’s^20^ study of adults over 21 years, only 1% of HUs continued high use for 2 consecutive years. High usage rates in the young population are worrying, not only because they incur societal costs, but because the high use of healthcare services may also have significant individual impacts. Persistent high use may exert negative effects on the development of personality and identity, as well as on one’s professional and social career. For example, it is possible that high healthcare use may reinforce personality traits that are known to be associated with greater use of services.^34^

In our data, one-third of adolescents who belonged to the HU group for at least 1 year were identified for becoming persistent HUs. However, there were also adolescents whose high use ended after 1 or 2 years. These findings regarding turnover rate are in line with those previously reported in the literature. For example, in the study of Vedsted et al,^32^ 40% or less of frequent attenders were FAs also the following year. Thus, the continuation of HU can be considered to be high, and single-year HU status is already an important indicator for persistent high use of services. Thus, special recognition and preventive actions should be applied to those persons with HU, because they pose elevated risk for pHU. However, more research is needed to recognize specific early factors such as the nature of the disorder and psychosocial status, which could indicate increased risk for persistent use.

In the analysis of attendance days with recorded ICD-10 diagnosis, we compared the prevalence of somatic and psychiatric morbidity between pHUs and non-pHUs according to main categories of ICD-10 diagnoses. These results were aimed to provide preliminary insight to the persistent high-utilizer phenomenon in health care services of young persons. Therefore, our findings can be contrasted to prior research on concerning diseases of mental, neurological, and musculoskeletal system. In our study, the pHU was particularly emphasized in diagnostic category for mental and behavioral disorders (RR = 4.46) This which result was in concordance with previous research showing that mental disorders have been the most common diagnoses among HUs and pHU patient groups among adolescents.^12,22^ This is understandable because mental disorders are mostly chronic in nature, and their treatment often requires regular therapy and follow-up visits over the long term. Moreover, in our study diseases of the nervous system were common among pHUs (RR = 3.39), which have also been reported as common in previous studies concerning persistent high use.^12,22^ Furthermore, diseases of the musculoskeletal system and connective tissue (RR = 3.23) emerged in the pHU group. This finding is in line with the study of Punjabi et al,^22^ in which they reported musculoskeletal disorders and cancers to be more frequent than mental health conditions as a primary diagnosis among high users. High prevalence of musculoskeletal disorders among adolescents is a worrying phenomenon, because it may indirectly indicate an impaired physical condition and reduced physical activity among young people nowadays.^13,28^ This is important to note because musculoskeletal problems are potential targets for prevention by various social means; for example, by improving hobby opportunities, and increasing physical activity in schools.

In Finland, a statutory assessment of the need for care must be carried out before every health-care visit. This assessment determines, among other things, whether the patient requires services. Based on the assessment, an evaluation of the urgency of care and the most appropriate professional to provide it is often made. This process typically relies on information provided by the parent or legal guardian and, whenever possible, on interviewing and even examining the child or adolescent. We fully recognize that need for services and actual service use do not always align; however, in Finland the problem is seldom overuse of services and more often underuse.^6,15^

In addition, communication between early childhood education centers, schools, and educational institutions and the health-care system is regulated—and in certain situations even mandated—by law when concerns arise. Failure to attend scheduled health services may itself trigger a need to investigate a child’s or adolescent’s situation more closely. Moreover, Finland employs a detailed coding system in health care to help identify, among other things, those in need of special support.

Unfortunately, register-based research cannot determine whether each provided service was in fact necessary; this would require reviewing patient records individually for each visit. However, the register data do include patient-record information for parents within the same family, enabling the examination—at the level permitted by register data—of how parental mental-health, substance-use, and addiction diagnoses relate to particular patterns of service use. This constitutes one of the planned aims for follow-up studies

When making international comparisons in the use of services, it is important to consider that the results between various countries may be affected by differences in how available services are and how they are organized. For example, there may be differences in the number of health examinations performed for varying age groups in the population. Also, there may be differences in morbidity from 1 country to another.^18^. In Finland, the healthcare system is based on public healthcare services to which everyone permanently residing in the country is entitled to obtain, which may increase the number of healthcare visits. In addition, the high number of visits among children and adolescents in Finland may be affected by annual health checks through school health care from the first to ninth grades.^14^ In Finland and other Nordic countries, vaccine coverage remains at a high level. In North Karelia, for example, coverage for measles, rubella, and mumps vaccinations has been slightly above the national average, at approximately 93.0%. In addition, the proportion of children who have received at least 1 dose of diphtheria, tetanus, pertussis, polio, or Hib vaccine has averaged around 98.7%.

Finnish healthcare is heavily regulated through national legislation, policy, and supervision by authorities like the Ministry of Social Affairs and Health (STM) and the National Supervisory Authority for Welfare and Health (Valvira). The system is also regulated by specific laws, including the Health Care Act and the Act on Organizing Healthcare and Social Welfare Services, which set standards for quality, safety, and evidence-based practices. Finland’s healthcare is a tax-funded, public system based on residency and is highly regulated, not insurance-based, although private services are also available and can be partially reimbursed through national health insurance (Kela).

In Finland, legislation requires that regular, age-specific health examinations for children and adolescents be conducted, and national statistical and supervisory authorities monitor the coverage of these examinations. In addition, several other monitoring statistics are available. For example, in North Karelia, the number of school healthcare physician visits per 1000 children aged 7 to 18 years was slightly lower (239.6) during the study period than the national average (267.2). However, the proportion of fourth- and fifth-grade pupils who had visited a school nurse at least twice during the school year was the same in North Karelia (44%) as the national average (44%). Overall, In Finland service systems and the availability of health and social care services are similar in all welfare counties. Therefore, the results of this study can be generalized especially to the Finnish children and adolescent population. The results of this study can be considered generalizable also to other Nordic countries because the healthcare systems in them are very similar.^17^

## Strengths and Limitations

A strength for this study is that the study population consists of 1 age group, so everyone has had the same regular visits in school healthcare. Moreover, because the study population is from the same wellbeing services county, people had the same services available during follow-up. The study population is well represented by the entire age cohort. Those who did not live in North Karelia for the entire 5-year follow-up period were excluded from the analysis, but they made up only a very minor section of the population.

The more detailed analyses of the study were performed on those attendance days that included an ICD-10 diagnostic code. The reasons for visits were coded exclusively with *kvist*, which nurses use to register reasons for visits only when an ICD-10 diagnosis is unavailable, they were not analyzed because the ICD-10 codes as primary diagnosis are set by physicians, and the criteria are clearer and more precisely defined than in ICPC-2. This also allows more homogenous data and is therefore a strength in our study. Our visit data does not include private sector visits, which, however, are probably relatively few in Finnish practice. We analyzed attendance days, and there could have occurred several visits on the same day to different professionals, of which only physician-diagnosed visits were included in the analyses of Table 1. Prevalence of diseases were analyzed in the main ICD-10 categories, which remains open, whether visit was related to chronic or acute conditions. Further, the role of multimorbidity a study participant belongs to 2 or more illness categories) to persistent HU-use remains unresolved.

## Conclusion

One-fifth of the entire population of adolescents born in 2004 was HU in at least 1 year, and one-third (34.7%) of all single-year HUs met further criteria for pHU when analyzed over the 4-year follow-up period. Being single-year HU is an important sign of elevated risk for persistent high utilization. In the pHU group, chronic diseases, and especially mental disorders, were highlighted, which shows that patients are receiving the treatment they need. More research on adolescents’ high utilization and its continuation is needed. It may thus be possible to find ways to reduce persistent high healthcare use among young people.

Our planned follow-up research will involve a more detailed analysis of data on the prevalence of chronic and acute disease groups, supplemented with additional information available from patient records—such as details of treatment content, family service use, and other relevant background factors. Given the breadth of the topic, this work will, for practical reasons, be presented in future articles of the research group.

## Supplemental Material

sj-docx-1-jpc-10.1177_21501319261421476 – Supplemental material for Prevalence of Adolescents’ Persistent High Utilization of Outpatient Healthcare Services and ICD-10 Diagnoses: A Retrospective 4-Year Population-Based Register Study Supplemental material, sj-docx-1-jpc-10.1177_21501319261421476 for Prevalence of Adolescents’ Persistent High Utilization of Outpatient Healthcare Services and ICD-10 Diagnoses: A Retrospective 4-Year Population-Based Register Study by Pipsa Lahtinen, Mika Niemelä, Helinä Hakko, Sanni Penttilä, Petri Kivinen and Sami Räsänen in Journal of Primary Care & Community Health
