# Supplementary material for: Prevalence of Adolescents’ Persistent High Utilization of Outpatient Healthcare Services and ICD-10 Diagnoses: A Retrospective 4-Year Population-Based Register Study
Source: J Prim Care Community Health. 2026 Feb 21;17:21501319261421476. doi: 10.1177/21501319261421476 (PMC12924922; doi:10.1177/21501319261421476)
Supplement: sj-docx-1-jpc-10.1177_21501319261421476 – Supplemental material for Prevalence of Adolescents’ Persistent High Utilization of Outpatient Healthcare Services and ICD-10 Diagnoses: A Retrospective 4-Year Population-Based Register Study [file sj-docx-1-jpc-10.1177_21501319261421476.docx]

**SUPPLEMENTARY MATERIAL**

**Supplementary Table 1.** Total and annual distribution of attendance days among adolescents born in 2004 (n=1483) during the period 2018–2021.

|  | **Number of visits and contacts to healthcare** | **At least one attendance day,**  **n (%) of persons** | **Sum of attendance days** | **Percentile distribution of**  **attendance days** | |
| --- | --- | --- | --- | --- | --- |
|  |  |  |  | **50_th_ percentile (median)** | **75^th^ percentile (upper quartile)** |
| Year: 2018 | 15143 | 1393 (93.9) | 13901 | **7** | **12** |
| Year: 2019 | 16045 | 1410 (95.1) | 14573 | **7** | **12** |
| Year: 2020 | 15253 | 1434 (96.7) | 13673 | **6** | **11** |
| Year: 2021 | 17409 | 1463 (98.7) | 15700 | **7** | **13** |
| Total | 63850 |  | 57847 |  |  |
| Cut-off point for high utilizer (HU) of health care | | | | | **12** |

Note: Attendance days = number of individual calendar days with contact to health care services.
